# *Foxf2*: A Novel Locus for Anterior Segment Dysgenesis Adjacent to the *Foxc1* Gene

**DOI:** 10.1371/journal.pone.0025489

**Published:** 2011-10-13

**Authors:** Richard McKeone, Helena Vieira, Kevin Gregory-Evans, Cheryl Y. Gregory-Evans, Paul Denny

**Affiliations:** 1 MRC Mammalian Genetics Unit, Harwell, Oxford, United Kingdom; 2 Department of Cell and Molecular Biology, Faculty of Medicine, Imperial College London, London, United Kingdom; 3 Department of Ophthalmology and Visual Sciences, University of British Columbia, Vancouver, British Columbia, Canada; Institut Jacques Monod, France

## Abstract

Anterior segment dysgenesis (ASD) is characterised by an abnormal migration of neural crest cells or an aberrant differentiation of the mesenchymal cells during the formation of the eye's anterior segment. These abnormalities result in multiple tissue defects affecting the iris, cornea and drainage structures of the iridocorneal angle including the ciliary body, trabecular meshwork and Schlemm's canal. In some cases, abnormal ASD development leads to glaucoma, which is usually associated with increased intraocular pressure. Haploinsufficiency through mutation or chromosomal deletion of the human *FOXC1* transcription factor gene or duplications of the 6p25 region is associated with a spectrum of ocular abnormalities including ASD. However, mapping data and phenotype analysis of human deletions suggests that an additional locus for this condition may be present in the same chromosomal region as *FOXC1*. DHPLC screening of ENU mutagenised mouse archival tissue revealed five novel mouse *Foxf2* mutations. Re-derivation of one of these (the *Foxf2* ^W174R^ mouse lineage) resulted in heterozygote mice that exhibited thinning of the iris stroma, hyperplasia of the trabecular meshwork, small or absent Schlemm's canal and a reduction in the iridocorneal angle. Homozygous E18.5 mice showed absence of ciliary body projections, demonstrating a critical role for *Foxf2* in the developing eye. These data provide evidence that the *Foxf2* gene, separated from *Foxc1* by less than 70 kb of genomic sequence (250 kb in human DNA), may explain human abnormalities in some cases of ASD where *FOXC1* has been excluded genetically.

## Introduction

Anterior segment dysgenesis covers a spectrum of disorders affecting the iris, cornea, trabecular meshwork and Schlemm's canal of the eye, which can result in abnormal aqueous humor drainage from the eye leading to raised intraocular pressure and glaucoma [1]. These abnormalities result from a primary defect in the migration and differentiation of neural crest cells that contribute to the development of the anterior segment structures [2]. Malformation of tissue specifically at the iridocorneal angle (iridogoniodysgenesis anomaly) or in the anterior stroma of the iris - contribute to the glaucoma phenotype [3], [4].

Anterior segment dysgenesis (ASD) phenotypes are inherited as autosomal dominant traits with variable expressivity and incomplete penetrance, pointing to a complex etiology [5], [6]. Nine different human genes have been associated with ASD or congenital glaucoma including *FOXC1, PITX2, PITX3, FOXE3, PAX6, MAF, CYP1B1* and *LMX1B*. Mutations in the *FOXC1* gene [7], or dosage effects due to deletions [8] or duplications [9], [10] in the 6p25 region that surrounds *FOXC1* can all cause iridogoniodysgenesis; as can mutations in the *PITX2/RIEG1* gene [11]. Patients with *FOXC1* mutations have a milder average prognosis for glaucoma development than do patients with any one of the known *PITX2* mutations [12]. One common link between these genes, other than their expression in the neural crest cells of the periocular mesenchyme [13], [14]; is that their upregulation can be triggered by Tgfb2 activity. Inactivation of this growth factor in mouse neural crest cells results in malformed trabecular meshwork, ciliary body and corneal endothelium cells [15].

Genetic evidence suggests that other genes near *FOXC1* may also be involved in the underlying etiology of iridogoniodysgenesis and other eye abnormalities associated with glaucoma. For example, deletion of 6p24-p25 proximal to the *FOXC1* locus causes anterior segment abnormalities [16], [17], [18]. Recombination mapping in families linked to 6p25 excluded *FOXC1* as the causative gene [19]. Furthermore, a patient with an unbalanced translocation between 6p25 and 4p14 was disomic for *FOXC1* but may have been monosomic for *FOXF2* [20].

To investigate whether the nearby *Foxf2* gene could be involved in anterior segment development and dysgenesis we took advantage of an ENU mutagenised DNA archive [21], [22], that allowed recovery of identified *Foxf2* mutant lineages. We describe the genetic analysis of an identified *Foxf2* mutation and the phenotypic features of the affected animals. These analyses suggest *Foxf2* is essential for normal anterior segment development, and that the *FOXF2* gene should be considered as an additional candidate for anterior segment dysgenesis in humans.

## Results

### Identification of *Foxf2* sequence variants from archival DNA

**Figure 1:**
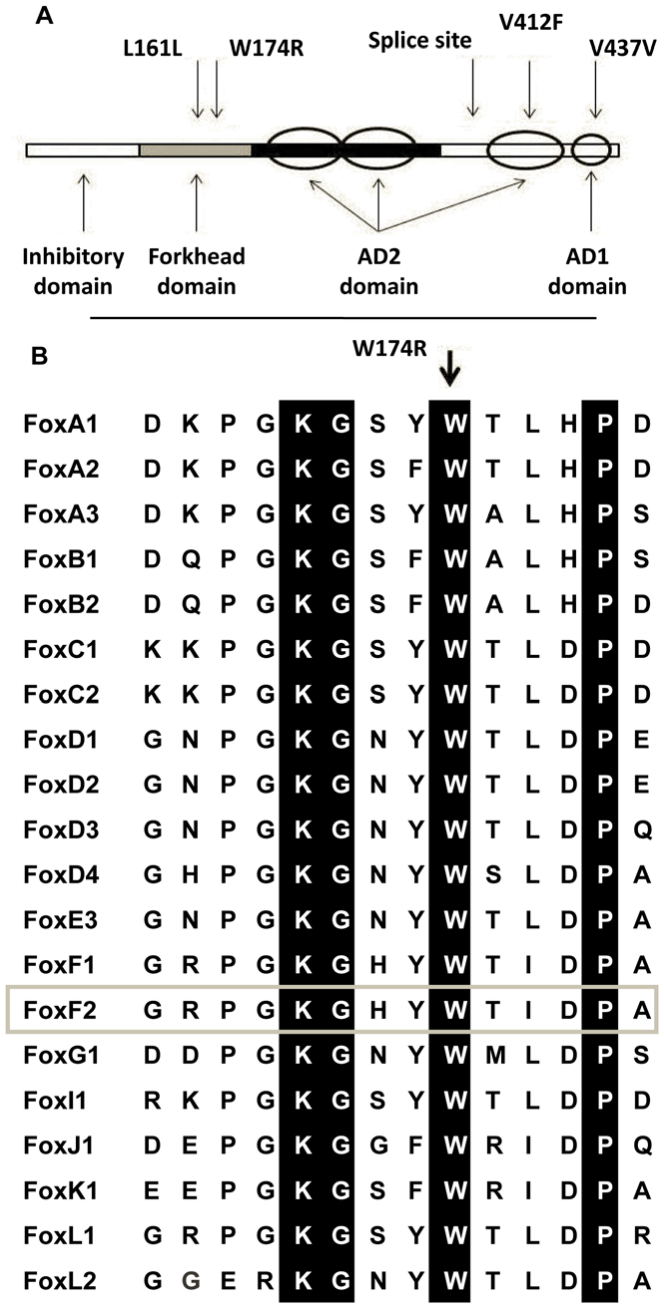
Foxf2 structural organization. **A**, Protein domains of Foxf2 with mouse mutations identified in the DHPLC screen. The domain structure is shown as described for mouse [23] including two activation domains at the 5′ end, but overlayed by the activation domain structure that was described for the human gene [49] with AD2 synergistic sub-domains (ovals) and AD1 domain (circle). **B**, The tryptophan codon at position 174 is conserved in the forkhead domain of all Fox proteins, of which several examples are shown. Foxf2 is highlighted by the grey box. Conserved residues are highlighted in black.

Archival DNA from tail biopsies of the F1 progeny of mice that had undergone ENU mutagenesis, was screened by DHPLC analysis followed by sequencing of samples that produced heteroduplexes. This protocol identified 5 sequence variants in the *Foxf2* genomic DNA (Table 1). Two base changes did not alter amino acid sequence and are therefore silent variants. Individual mouse GSK 14H3 carried a T→A transversion at position 821 of the *Foxf2* transcript (Figure 1A). This change results in a W174R amino acid substitution in the forkhead DNA binding domain of the protein. In mouse MRC 18C1, a G→T transversion at position 1535 of the *Foxf2* transcript resulted in a conservative V412F amino acid substitution in the third sub-region of the AD2 transactivation domain [23]. An A→G transition was identified in mouse MRC 31H8 at the third base of the intron. The six base region following the end of exons is generally highly conserved between eukaryotic 5′ splice donors, but this third base is the least conserved of these positions. In an analysis of intron – exon boundaries within 1446 genes, 35% of splice sites contain an adenosine at this position and 60% a guanosine, whereas all of the other positions showed much greater levels of conservation [24] - so interference with normal splicing could be considered unlikely. However, 106 disease associated A→G splice site mutations at the equivalent position (IVS+3) in the donor regions of 79 genes, are present in the human gene mutation database (HGMD) [25]. Thus, the possibility remains that this mutation could result in aberrant splicing.

**Table 1:** *Foxf2* sequence variations found in the ENU archival DNA.

Mouse ID	Transcript base position	DNA variant	Amino acid Change	Type of change
GSK1B11	784	G→T	L161L	Silent
GSK14H3	821	T→A	W174R	Non-conservative
MRC31H8	Intronic IVS1+3	A→G	N.A.	Possible aberrant splicing
MRC18C1	1535	G→T	V412F	Conservative
MRC25H1	1575	G→C	V437V	Silent

The *Foxf2* mutation rate within the ENU archives that was previously determined during the discovery of the *Foxf2* ^W174R^ mutation and one of the silent mutations [22] can now be updated to 5 mutations in 1340 bp of 7990 individuals.

### Recovery of the *Foxf2* ^W174R^ mouse lineage

Analysis of inter-species conservation, physico-chemical implications of the amino acid substitutions and the position of the mutations in the protein structure suggested that *Foxf2* ^W174R^ was the mutation that was most likely to disrupt the function of the gene product. The tryptophan residue is conserved in all genes with a forkhead domain (Figure 1B) and occurs within a β-sheet structure. This mouse line was therefore re-derived for further examination.

**Figure 2:**
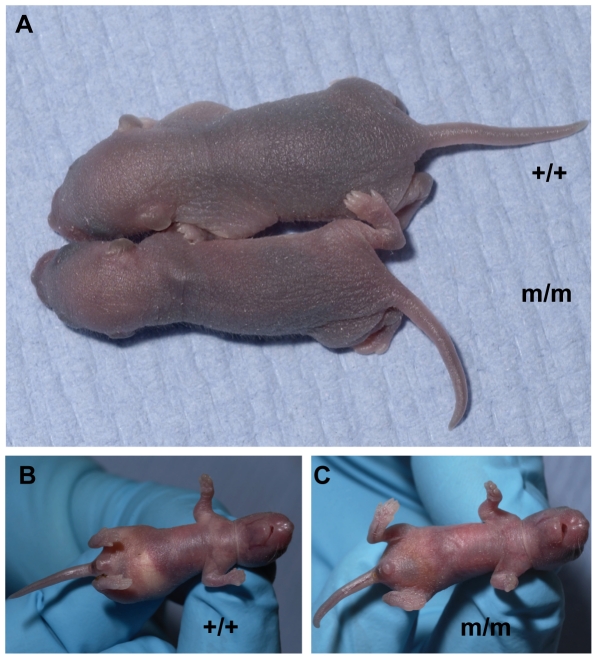
Phenotype of homozygous *Foxf2* ^W174R^ pup compared with wildtype. **A**, A noticeable difference in gross size can be seen by 3 days of age in mutant pup. **B&C**, In contrast to the homozygous knockout, the homozygous *Foxf2* ^W174R^ individuals appear normal at birth, although the amount of milk consumed (arrow) is reduced compared to wildtype littermates.

Homozygous mutants die within 14 days of birth and in 16 individuals, none showed evidence of malformation in either the primary or secondary palate. This is in contrast to earlier findings in the homozygous *Foxf2* knockout mice which die within 18 hours with cleft palates and gas distended guts [26]. *Foxf2* ^W174R^ homozygotes appear normal at birth but fail to thrive and by 3 days are noticeably smaller than their wildtype littermates (Figure 2). As in the knockout, microscopic analysis did not reveal any lung defects despite the gene's intense expression in the lung [23] which, in common with the eye but not any of the other tissues that express *Foxf2*, continues to express the gene into adulthood [27]. Heterozygous mice appear to thrive normally and are fertile, as was the case for knockout mice.

### *Foxf2* ^W174R^ eye phenotype analysis

**Figure 3:**
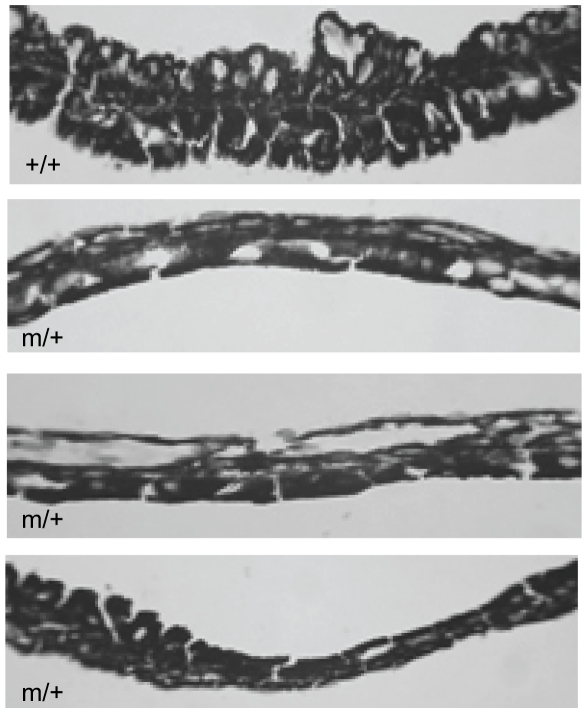
Iris phenotype of *Foxf2* ^W174R^ mice. The iris in wildtype mice (+/+) consists of a robust upper stromal layer (arrowhead) and a lower pigmented epithelium (arrow). The stroma in three heterozygous (m/+) mice is flattened or atrophic. The bottom image shows variable thickness across the iris tissue in both stromal and epithelial layers. Scale bar  = 40 µm.

**Figure 4:**
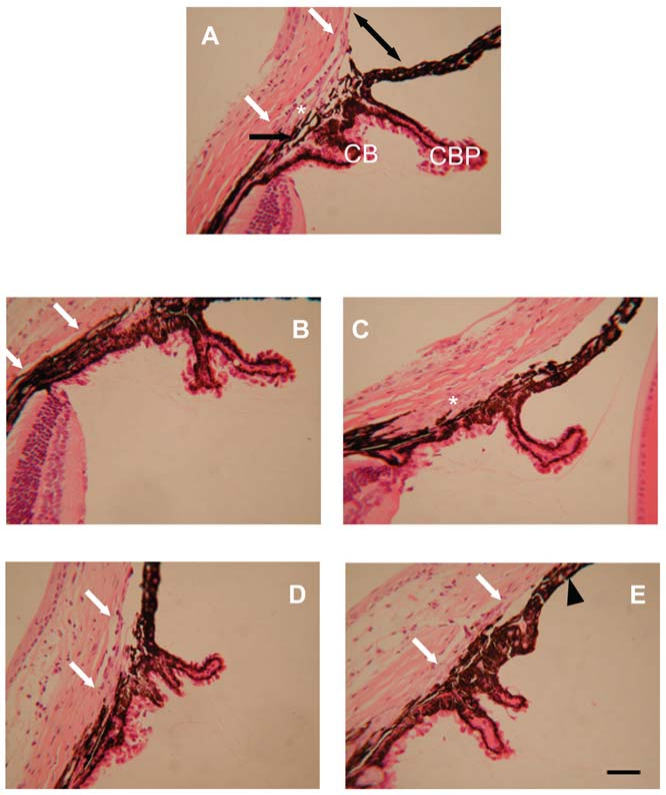
Iridocorneal phenotypic features in *Foxf2* ^W174R^ mice. (**i**), in wildtype mice Schlemm's canal extends between the two white arrows. The trabecular meshwork is indicated by an asterisk. The ciliary body (CB) and ciliary body process (CBP) extend into the anterior chamber. The ciliary muscle is indicated by the black arrow. Iris Normal iridocorneal angle (**A**) indicated by double-headed arrow. (**B–E**), phenotype in heterozygous *Foxf2* ^W174R^ mice. All mice had shortened or absent Schlemm's canal and a hypoplastic CB or CBP. (**C**), Trabecular meshwork is hypoplastic and the iris is parallel to the cornea (no angle). (**E**), Trabecular meshwork is hypoplastic and iridocorneal adhesion (arrowhead) is present. Scale bar  = 50 µm.

The eyes from ten *Foxf2* ^W174R^ heterozygous mice that were 45 days of age were examined by light microscopy. The iris stroma showed irregular thinning of the tissue (compared to wildtype (Figure 3)) and a loss of structural organization. A number of unusual features were observed in the irido-corneal angle of all mice analysed (Figure 4). The canal of Schlemm was smaller in most of the mice (7/10) and was not seen at all in others (2/10); the trabecular meshwork showed signs of hypoplasticity (7/10); one individual had a hypoplastic ciliary muscle. In some mice the angle between the cornea and iris was significantly reduced (6/10) and in one individual the two tissues were adherent. The phenotype variability that was seen between different animals was also apparent between the eyes of individuals, although to a lesser degree. This type of variation is also seen in *Foxc1* heterozygous mice [28] as well as in human disease [29] and may be dependent on genetic background. Although this variability could be attributed to genetic modifiers it is also likely to be influenced by the presence of normal and abnormal tissue, probably reflecting stochastic events in which the spatiotemporal regulation of *Foxf2* downstream targets is critical to anterior segment development. Nevertheless, all mice exhibited two or more defects. Histological analysis showed no signs of damage to the cornea, optic nerve or retinal nerve fibres at 45 days of age (data not shown).

**Figure 5:**
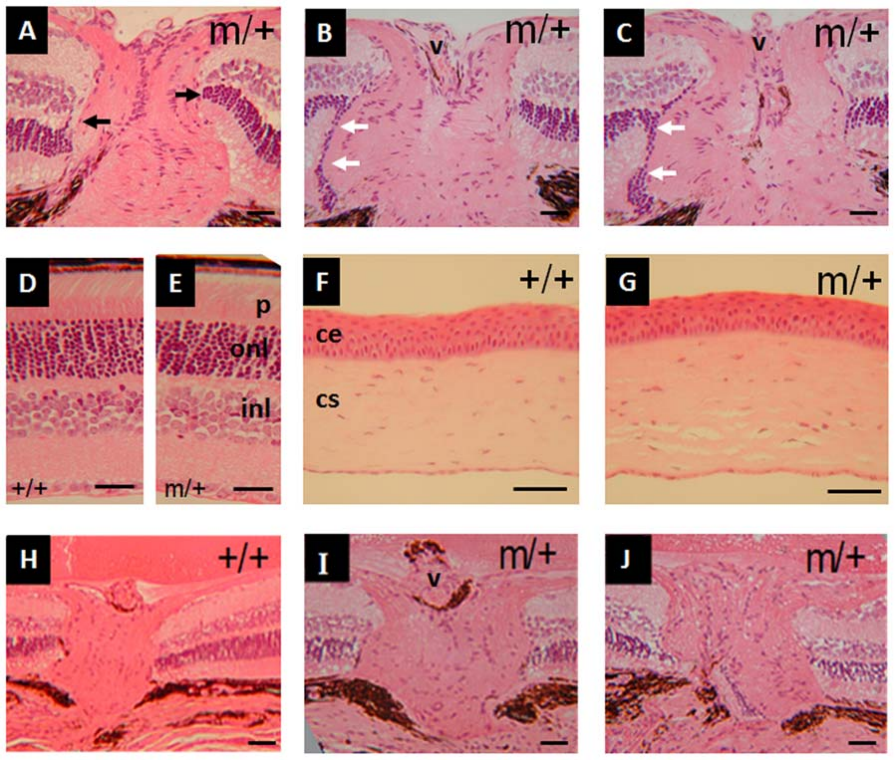
Representative images of eye phenotype of aged heterozygous *Foxf2* ^W174R^ mutant mice. **A**, 6 month old *Foxf2* ^W174R^ eye (m+/) showing normal retinal termination at the edge of the optic nerve fibre layer (black arrows). **B&C,** sections through two other 6 month old *Foxf2* ^W174R^ eyes showing abnormal bulge in the nerve fibre layer. The outer nuclear layer of the retina can be seen to continue at the surface of this bulging tissue (white arrows). **D&E**, retinal sections in 6 month eyes. **F&G**, corneal sections in 6 month eyes. **H–J**, 12 month *Foxf2* ^W174R^ optic nerves that do not show signs of glaucomatous change. v, vein; p, photoreceptors; onl, outer nuclear layer; inl, inner nuclear layer; ce, corneal epithelium; cs, corneal stroma. Scale bar  = 50 µm.

To investigate the effect of the W174R mutation in older mice (6 months), the retina, cornea and optic nerve of heterozygous mice were examined to determine if there was any apparent glaucomatous damage. Histological analysis of 18 mice showed a range of anterior segment defects as previously seen in younger mice. In addition two mice appeared to have bulging eyes that can be associated with raised intraocular pressure. However, on histological investigation there appeared to be extraneous amorphous tissue between the retina and the lens. Histological analysis in the majority of *Foxf2* ^W174R^ heterozygous mice (16/18) revealed no substantial damage to the optic nerve, retina or cornea (Figure 5). In two mice there was swelling of the optic nerve, which disrupted the outer nuclear layer of the retina (Figure 5B&C). In mice that were 12 months of age, the optic nerve appeared to be normal because there was no optic nerve cupping as would be expected at this age if glaucomatous damage had occurred [30].

**Figure 6:**
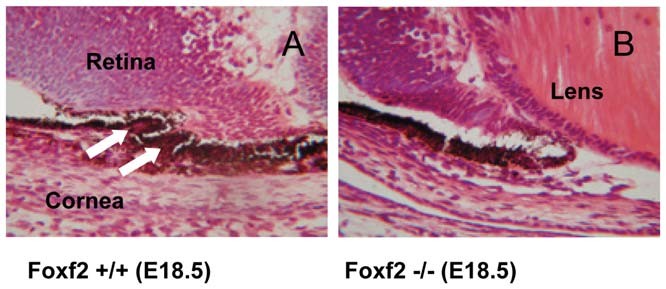
Comparison of iridocorneal development in homozygous *Foxf2* ^W174R^ embryos. **A**, The ciliary body is beginning to form in E18.5 wildtype embryos, showing two finger-like projections of evaginated tissue (white arrows). **B**, Formation of the ciliary body is absent in the E18.5 homozygous *Foxf2* ^W174R^ mutant mouse eye.

To investigate whether homozygote *Foxf2* ^W174R^ embryos displayed iridocorneal defects, E18.5 embryos were examined by histology. Evagination of tissue from the anterior optic cup begins at E14 to form the future iris and ciliary body. At E18.5 finger-like projections of tissue forming the ciliary body processes are clearly visible in wildtype littermates (Figure 6A). However, there was no evidence of tissue evagination in the homozygote embryos (Figure 6B). Heterozygous mice at this stage appear indistinguishable from wildtype mice.

Mouse subjects were from several divergent lineages that had been outcrossed to between G5 and G8 from the single mutagenised founder. This meant that the likelihood that the observed phenotype resulted from mutations in ASD associated genes on other chromosomes was negligible. However, due to the close genetic linkage of *Foxc1* to *Foxf2* and the similarity between the *Foxf2* ^W174R^ and *Foxc1* mutant and knockout eye phenotypes, it was important to ensure that no mutations in *Foxc1* were responsible for the observed phenotype. The *Foxc1* coding sequence of the *Foxf2* ^W174R^ mouse was therefore sequenced. No differences between this sequence in the *Foxf2* ^W174R^ mouse and the *Foxc1* mouse reference sequence were present.

## Discussion

Chromosome 6p25 is a major locus for anterior segment dysgenesis (ASD). Previous reports of cytogenetic abnormalities are consistent with the notion that the eye is exquisitely sensitive to both reduced and increased dosage in this chromosomal region. Although *FOXC1* dosage is a major contributor to eye defects localised to this region, we now provide evidence that *Foxf2* is a novel locus for anterior segment dysgenesis. Heterozygous mutation of the forkhead binding domain of the *Foxf2* gene is associated with anterior segment defects in the iridocorneal angle of mice, whereas homozygous defects are lethal. At E18.5 the development of the ciliary body is defective, suggesting that *Foxf2* is essential for normal ciliary body formation. These data support the role of *Foxf2* in normal anterior segment development.

Data from the characterisation of a 200 kb deletion located 1.2 Mb upstream of *FOXC1* [31] suggested that mutations could induce a phenotype via long-range effects. It is therefore feasible that the observed phenotype in *Foxf2W174R* individuals could be the result of a mutation within a *Foxc1* regulatory region. However, other evidence to support the involvement of *Foxf2* in anterior segment dysgenesis, including the patterning of its ocular expression [27], [32], the high level of conservation and physico-chemical changes of the mutagenised amino acid and the absence of *Foxc1* coding mutations; in combination with the observed physical phenotype - all contribute towards a greatly strengthened candidacy of *Foxf2*.

Previous studies have shown that targeted deletion of *Foxf2* caused palate malformations and an abnormal tongue [26]. Analysis of *Foxf2* knockout mice subsequently revealed megacolon, colorectal muscle hypoplasia and agangliosis [33]. However, the colon was not analysed in the present study and therefore the effects of this mutation on the gut would seem like a promising focus of future investigations into the effects of the *Foxf2* ^W174R^ mutation. *Foxf2* is expressed in the absence of its closest paralogue (*Foxf1*) in the CNS, ear, and limb buds as well as the eye [27] so these systems are also worth prioritising in the search for other potential *Foxf2*-associated phenotypes.

The effect on eye development was not examined in previous analyses of *Foxf2* knockouts [33]. Interestingly however, one study did demonstrate normal *Foxf2* expression in the periocular mesenchyme of the developing eye at about E12.5 [32]. Furthermore, *in situ* hybridisation established that there was continued *Foxf2* expression from E13 through to adult stages [27]. High levels of *Foxf2* expression at E17 were observed in the developing ciliary body and choroid. These data support the abnormal morphological finding in the developing ciliary body in homozygous *Foxf2* ^W174R^ embryos.

The difference in phenotype that was identified between targeted knockout and homozygous missense mutation could suggest that *Foxf2* ^W174R^ is a hypomorphic allele, However it is also possible that the differences are due to genetic background and that the mutation causes a complete loss of function. Molecular modelling of *FOXC1* in a previous study revealed that a tryptophan residue (Trp152) - the direct homologue of Trp174 in *Foxf2*, is one of nine critical intramolecular interaction residues that maintain structural integrity of the forkhead winged helix structure [34]. It therefore seems likely that disruption of Trp174 in *Foxf2* would lead to protein instability. Another example of an unstable forkhead transcription factor with a mutation in the DNA binding region is the I87M variant of *FOXC1* [34]. Cos7 cells transfected with this mutant plasmid demonstrated markedly reduced levels of the protein at only 5% of levels observed for the wildtype, but the molecule retained its nuclear localisation function. A drastic reduction but not complete destruction of protein functionality, could explain the reduced severity of phenotype that is observed in association with the *Foxf2* ^W174R^ mutation and would be consistent with the hypothesis that haploinsufficiency plays a key role in the pathogenesis of Fox associated anterior segment anomalies.

The ocular abnormalities found in *Foxf2* ^W174R^ mice are variable in eyes from different individuals, recapitulating the variable expressivity observed in human patients with ASD. Schlemm's canal was often smaller than typically seen in wild-type eyes and trabecular meshwork was either missing or was underdeveloped, suggesting abnormal migration of mesenchymal cells into the iridocorneal angle. The ciliary body malformations may affect aqueous humor production and secretion of antioxidant proteins into the aqueous humor [35]. Aqueous humor is drained through the trabecular meshwork, therefore alterations in aqueous humor homeostasis are likely to occur when these tissues are malformed and could contribute to changes in intraocular pressure [36]. The iridocorneal abnormalities observed in the *FoxF2* ^W174R^ mice are very reminiscent of those seen in mice that are heterozygous for *Foxc1* or *Foxc2* mutations [28]. Since *Foxc1*, *Foxc2* and *Foxf2* are all expressed in the developing periocular mesenchyme, this suggests that this tissue is particularly sensitive to gene dosage [9], [37].

Despite the high level of conservation in their DNA binding domain, forkhead transcription factors are an extraordinarily diverse group of genes with roles as varied as development, homeostasis, stress response and cell cycle control [38]. Intriguingly, mutations in a number of forkhead genes can result in a variety of disorders affecting the eye. Mutations of the *FOXE3* gene affect lens development and can be inherited as either an autosomal dominant or recessive trait [39]. The more severe recessive trait is associated with bilateral microphthalmia, aphakia, corneal defects and glaucoma, whereas the milder autosomal dominant trait is associated with iris hypoplasia, Peters' anomaly, and isolated cataract. Mutations of the *FOXC2* gene cause lymphedema-distichiasis syndrome [40] - characterised by double rows of eyelashes, ptosis, photophobia and anterior segment anomalies reminiscent of those caused by *FOXC1* [7]. *FOXL2* mutations cause blepharophimosis-ptosis-epicanthus inversus syndrome (a complex eyelid malformation) [41] and in some patients lacrimal duct anomalies, amblyopia, strabismus, and refractive errors. In addition, expression of three other forkhead genes; *Foxg1* [42] *, Foxd1* [43] and *Foxn4* [44], has also been shown in the developing retina. It is clear that forkhead transcription factors play a critical role in the developing eye, and now the *Foxf2* gene can be added to this growing list.

The 6p25 region contains a forkhead cluster (*FOXC1/FOXF2/FOXQ1*) in which *FOXC1* is separated from *FOXF2* by less than 250 kb of genomic DNA, and *FOXQ*1 is 470 kb proximal of *FOXC1*. Because duplication and deletions of this region in human disease often contain more than one of these genes, confirmation of pathogenicity has relied on specific mutations in animal models. Although gene knockouts [28], [45] and naturally occurring mutations [14] recapitulate *FOXC1* deletions or mutations, no model carrying an additional functional copy of *FOXC1* has been developed to explore gain-of function effects seen in interstitial gene duplication events. Since our data provides evidence that *Foxf2* in mice is also critically involved in anterior segment development, then duplications or deletions containing both *FOXF2* and *FOXC1* in patients may contribute to the phenotype. This is supported by clinical observations where interstitial duplication of *FOXC1* alone causes an iris hypoplasia phenotype, whereas duplications containing both genes (plus several others depending on the extent of the duplication) cause microcornea and ptosis, without iris hypoplasia [46]. This suggests that different combinations of transcription factor gene dosage within cytogenetic abnormalities influence how eye development is affected.

## Materials and Methods

### DHPLC mutation scanning

We used ENU archival DNA that was generated as previously described [21], [22] as a template for *Foxf2* mutation scanning using DHPLC [47]. DNA concentrations were determined with a Spectramax 190 spectrophotometer (Molecular Devices). Five of six overlapping sets of primers were used for amplification of *Foxf2* (Table 2). For each PCR reaction, 10 ul of pooled archive DNA (4 samples) was added at a concentration of 5 ng/µl. Following amplification of the DHPLC targets, thermal cycling using the WAVE™ DNA Fragment Analysis System (Transgenomic, Cheshire, UK), was used to denature and then re-anneal PCR products with the following parameters: 95°C for 4 min, 45 cycles of 93.5°C for 1 min with a reduction of 1.5°C per cycle down to 25°C.

**Table 2:** Primers used for the gene driven screen and mouse *Foxf2* sequencing.

Primer Set	Forward	Reverse	Product Size
Exon1a	CTCGCCCGATTTGTGGAC	AGCGCGATGTACGAGTAAGG	414
Exon1b	AGTGGAGGCACCAAGAAGG	GGAACGAACCCTCCTCAAAC	316
Exon1c	TTCCCCTTTTTCCGTGGCGC	TGGCCATATAGGTGGAGCCC	460
Exon1d*	TCAAGGCGGTTATGGTGGCC	AGAGGCTCTCAGAGGCTCCG	513
Exon1e*	ACACCACCTCCACCACCAC	AGAGGCTCTCAGAGGCTCCG	433
Exon2a	AGCTGCCTTTACACCCTCAG	ACAGTGTGAGTCCGTTGCAG	384

Primer sets Exon1a-1e were used to screen the first exon and flanking regions, primer set Exon2a was used to screen exon 2. * Primer set Exon1e replaced Exon1d for the screen of the MRC archive.

### Sequencing

PCR amplification products from pooled DNA that exhibited evidence of heteroduplexes in their DHPLC profiles, were individually PCR-amplified and screened by DHPLC. The single DNA heteroduplex that was identified was sequenced on both strands to determine the mutation. PCR products were purified using a QIAquick PCR purification kit (Qiagen) and sequencing was carried out using BigDye 3.1 terminator chemistry on an ABI prism 377 DNA sequencer. Sequences were aligned and compared with consensus data obtained from the mouse genome database (http://genome.ucsc.edu).

### Mutant mouse recovery and genotyping

Recovery of the mutant mouse lineage was achieved by *in vitro* fertilisation with archival sperm and C3H/HeH females using standard methodology. Genotyping of the *Foxf2* ^W174R^ mice was performed by *Sfc*I (which cuts the mutant locus) and *Bsr*I (wildtype locus) restriction digestion of the exon1c PCR product to distinguish *Foxf2* ^W174R^ heterozygotes from homozgotes and wildtypes. Because the C3H mice carry a *Pde6b* ^rd1^retinal mutation affecting the eye, the identified *Foxf2* ^W174R^ mice were outcrossed to C57BL/6 mice for 2 generations. To exclude *rd1* carriers, genotyping was performed with the following two primers F: 5′-ACCTGAGCTCACAGAAAGGC-3′ and R: 5′-GCTTCTAGCTGGGCAAAGTG-3′ as described previously [48]. The mutation was detected by *Dde*I restriction digest (which cuts the *Pde6b^rd1^* mutant locus) and *Snab*I (wildtype locus) thus allowing differentiation between *Pde6b^rd1^* heterozygotes, homozygotes and wildtypes. All subsequent analyses were carried out on mice with only the *Foxf2* mutation. Primers for sequencing the *Foxc1* gene are in Table 3. All animal work was carried out in accordance with the UK Animals (Scientific Procedures) Act, 1986. The Harwell ethical committee approved the study and the work was performed under UK Home Office project licence numbers 30/1517, 30/2049 and 30/2228.

**Table 3:** Primers used for sequencing mouse *Foxc*1.

Primer Set	Forward	Reverse	Product Size
Foxc1_A	AGTCCTCGCCTGGGTGAC	CTCGCAGCCCACTCAGTTC	401
Foxc1_B1	AGTTGATCCGAACGTTCCTC	GCGCGATAGTAGCTCTGCTC	387
Foxc1_B2	GCGCTACTCGGTGTCCAG	CTTCTTGTCCGGGGCATTC	286
Foxc1_C	GAAGCCGCCCTACAGCTAC	CTCTCGATTTTGGGCACTG	552
Foxc1_D	AAGACGGAGAACGGTACGTG	CTGCAGGTTGCAGTGGTAAG	528
Foxc1_E	CAGCCAGAGCTCCAGTGC	CGTGCGGTACAGAGACTGAC	532
Foxc1_F	GGATCGGCTTGAACAACTCC	TCCCGTTCTTTCGACATAGG	540
Foxc1_G	CTTTCCTGCTCATTCGTCTTG	TTTGCAGAAAACGCTGTAGG	558
Foxc1_H	TGTCAAATTTCGCTAAACTCAG	TTTCCTGCCTTCTTACTCTTCC	600
Foxc1_I1	TTTGAAGACTTACAGCAATAACCAG	GTAATCAAACCGCCATCTCC	240
Foxc1_I2	TTAGGGTGATCTGCCCTGTC	TCCCTGGCTATTATGTTACCG	280

### Histological analysis

Eyes were enucleated and placed in 50% Karnovsky's fixative for 45 minutes. Eyes were then washed 3×30 min in PBS, dehydrated through a graded ethanol series (50%, 70%, 90% and 3 times in 100%) and then embedded in paraffin wax. Whole eye sections cut sagitally to a thickness of 5 µm were counterstained with hematoxylin and eosin. Retinal histology was imaged using a digital camera mounted on an Olympus 1×71 microscope.
